# Continuous surgical multi-level extrapleural block for video-assisted thoracoscopic surgery: a retrospective study assessing its efficacy as pain relief following lobectomy and wedge resection

**DOI:** 10.12688/f1000research.16857.1

**Published:** 2018-11-12

**Authors:** Mark Larsson, Anders Öwall, Ulrik Sartipy, Anders Franco-Cereceda, Barbro Johansson, Jan G. Jakobsson

**Affiliations:** 1 Department of Molecular Medicine and Surgery, Karolinska Institute, Stockholm, Sweden; 2 Function Perioperative Medicine and Intensive Care, Section for Cardiothoracic Anaesthesia and Intensive Care, Karolinska University Hospital, Stockholm, 17176, Sweden; 3 Heart and Vascular Theme, Karolinska University Hospital, Stockholm, 17176, Sweden; 4 Department of Anaesthesia Danderyds Hospital, Clinical Sciences Karolinska Institute, Stockholm, 18288, Sweden

**Keywords:** postoperative pain, VATS surgery, extrapleural block

## Abstract

**Background: **Video-assisted thoracoscopic surgery (VATS) causes less postoperative pain than thoracotomy; however, adequate analgesia remains vital. As part of a multi-modal postoperative analgesia, a continuous surgeon-placed extrapleural block catheter is an option. The aim of this retrospective study was to evaluate the analgesic efficacy of a continuous extrapleural block as part of a multimodal analgesic regimen after VATS in general, and VATS lobectomy and wedge resection in particular.

**Methods: **Case records for patients having undergone VATS surgery and been provided a multi-level continuous extrapleural block with an elastomeric pump infusing levobupivacaine 2.7 mg/ml at a rate of 5 ml/h during 2015 and 2016 were reviewed. Pain (Numeric Rating Scale) at rest and mobilisation as well as opioid requirement (daily, postoperative days 0-3, as well as accumulated) were analysed.

**Results: **In all, 454 records were reviewed: 150 wedge resections, 264 lobectomies and 40 miscellaneous cases. At rest, pain was mild median NRS rated 3-3-1-1 for postoperative day (POD) 0 to 3, during movement, pain was rated moderate during POD 0 and 1 and mild the remaining days (median NRS 4-4-3-3 for POD 0-3). The proportion of patients exhibiting mild pain at rest increased from 55% on POD 0 to 81 % on POD 3. The percentage of patients experiencing severe pain at rest decreased from 15% to 6%. Median oxycodone consumption was 10 mg per day for POD 1-3. Pain after VATS wedge resection was significantly lower at POD 1 and 3 compared to pain after VATS lobectomy.

**Conclusion: **We found a continuous surgeon-placed extrapleural catheter block to be a valuable and seemingly safe addition to our multimodal procedure specific analgesia after VATS. Whether the efficacy of the block can be improved by increasing local anaesthetic and/or adding adjuncts warrants further investigation.

## Introduction

Minimally invasive thoracic surgery by means of video-assisted thoracoscopic surgery (VATS) has been shown to cause less postoperative pain than thoracotomy ^1– 4^. Even though it is not as painful, VATS still requires adequate postoperative analgesia. As part of a multimodal analgesic regimen, different techniques using local anaesthetics can be used to block pain impulses from the surgical area. Epidural, paravertebral and intercostal blocks are all possible modalities. Compared to epidural block, using continuous extrapleural, paravertebral or intercostal block is thought to offer a more limited thoracic block, with possibly fewer side-effects.

For thoracotomy, several prior studies, including a Cochrane systematic review from 2016, all conclude that epidural and paravertebral block offer similar pain relief, with a paravertebral block possibly having fewer complications ^5– 8^. The benefit vs. risk for the paravertebral technique has, however, been argued ^9^.

The optimal pain management approach following elective VATS is still not known. A review by Steinthorsdottir *et al.* ^10^ could not provide any firm recommendation. No firm conclusion could be drawn assessing available evidence around thoracic epidural, multilevel and single paravertebral, paravertebral catheter, intercostal catheter, interpleural infusion and long thoracic nerve block. The most recent study comparing epidural and percutaneous paravertebral block by Kosiński *et al.* ^11^ showed that the paravertebral continuous block technique was a feasible and safe alternative to epidural analgesia. Hutchins *et al.* ^12^ found ultrasound-guided continuous paravertebral catheter to provide prolonged pain control and superior patient satisfaction compared with single-shot intercostal block after VATS.

In 2014, continuous extrapleural block after VATS was implemented as a standard technique for pain management following VATS surgery at Karolinska University Hospital.

The aim of the study was to assess the quality of pain treatment after VATS with the routine use of a multi-level continuous extrapleural block as part of a multimodal analgesic strategy.

## Methods

### Study background

This is a retrospective patient chart study. The study protocol was approved by the regional Human Research Ethics Committee, Stockholm, Sweden (Dnr. 2017/500-31). The need for informed consent was waived by the Ethics Committee. All patients who underwent VATS and received a continuous extrapleural block were included. Records for patients who received an extrapleural block between January 2015 and December 2016 were reviewed. Patient demographics, type of surgery, occurrence of post-operative nausea and vomiting (PONV) or other adverse events, and the postoperative pain course were reviewed and compiled. Data on other analgesics including opioids were also collected. If needed, opioid dose was converted to oral oxycodone equivalents (10 mg oral morphine considered equivalent to 5 mg oral oxycodone).

### Extrapleural catheter

During surgery, a multiply perforated 19-cm extrapleural catheter was inserted by the surgeon, under thoracoscopic control, in a posterolateral position parallel to the spine, thereby covering multiple intercostal spaces. An initial bolus of 75 mg levobupivacaine was followed by an additional bolus of mepivacaine 100 mg at the end of surgery. An elastomeric pump infusing levobupivacaine 2.7 mg/ml at a rate of 5 ml/h (13.5 mg/h) was connected to the catheter and subsequently continued for up to 3 days.

### Additional analgesia

Patients received oral slow-release paracetamol (665 mg, 2 tablets three times daily) and NSAID (naproxen, 250-500 mg twice daily) if tolerated. Patients received oral slow-release oxycodone twice daily with rescue short-acting oxycodone if needed.

### Pain score

Pain was assessed by Numeric Rating Scale (NRS) ^13, 14^. From the charts, we extracted the NRS score describing the most intensive pain at rest and during ambulation for each day from the day of operation (POD 0) to postoperative day 3 POD3.

For analysis, surgeries were categorised into two groups. In this report, we considered single and multiple wedge resections procedures causing similar trauma and postoperative pain. Similarly, lobectomy, bi-lobectomy and segment resection were considered as comparable procedures. Consequently, surgical procedures are reported as either a) wedge resection or b) lobectomy.

### Statistics

Demographics are presented as median and range. Pain is presented as median and range and further classified into categories; mild pain as NRS <4, moderate pain as NRS 4-7 and severe pain as NRS >7. For statistical analysis categorized pain was compared as mild versus non-mild (moderate and severe). After calculating the median opioid requirement, we categorized postoperative opioid need as either no opioid if none, low opioid dose if lower than the calculated median opioid requirement or high opioid dose if higher than the calculated median opioid requirement. Categorical data is presented as frequency and percent.

 Chi-square test was used for test of significant differences between pain categories, age groups, sex and procedure. Mann-Whitney U-test was used for test of significant differences in cumulative opioid dose for different age groups and procedure.

## Results

A total of 510 patients received an extrapleural catheter during the period studied.

**Figure 1.**
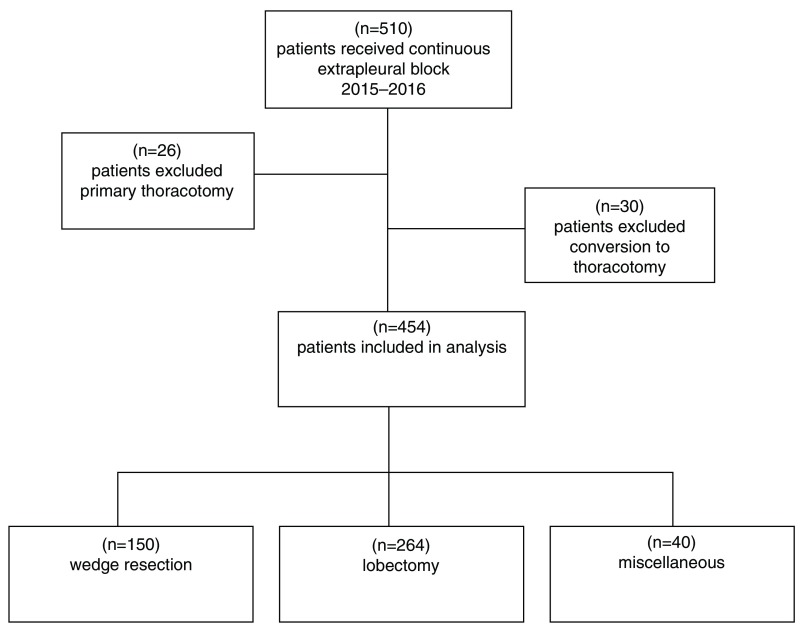
Patient inclusion flow chart.

A thoracotomy was performed as the primary procedure in 26 patients. The remaining 484 patients were scheduled for VATS procedures. In 30 cases VATS was converted to thoracotomy. Neither patients that underwent primary thoracotomy nor patients converted to thoracotomy are included in our analysis. Of 454 cases analysed, 150 procedures were classified as wedge resection, 264 cases as lobectomy and the remaining 40 cases as miscellaneous (thymus resection, extirpation of a nodulus, lymph node(s), cyst or hamartoma and decortication with or without wedge resection) ( Figure 1).

Baseline characteristics are presented in Table 1. All patients were followed in-hospital from day of operation (POD 0) until POD 3, except for two patients discharged on POD 1, and 37 patients discharged on POD 2, reducing the total number of pain assessments. Missing data occurred where we could not retrieve a pain score or opioid dose, either due to patient discharge or lacking data in the charts. We lack NRS scores with increasing frequency from POD 0 to POD 3.

**Table 1.** Demographics for all video-assisted thoracoscopic surgery (VATS) patients 2015–16, and for VATS wedge resection and lobectomy only.

Variable	All	Wedge resection	Lobectomy
Patients, n (%)	454	150 (33%)	264 (58%)
Age (years) *	68 (14-85)	66 (18-85)	69 (27-84)
Sex (f:m), n	271:183	85:65	167:97
Weight (kg) *	73 (36-129)	74 (45-125)	72 (36 - 129)
Chest drain (days) *	1 (0 -17)	1 (0.5 - 6)	1 (0 - 13)
Chest drain removed POD 1, % †	73	89	66

*Data given as median (range). †Percentage of patients where chest drain was removed by postoperative day (POD) 1.

## Pain at rest and during movement after VATS

**Figure 2.**
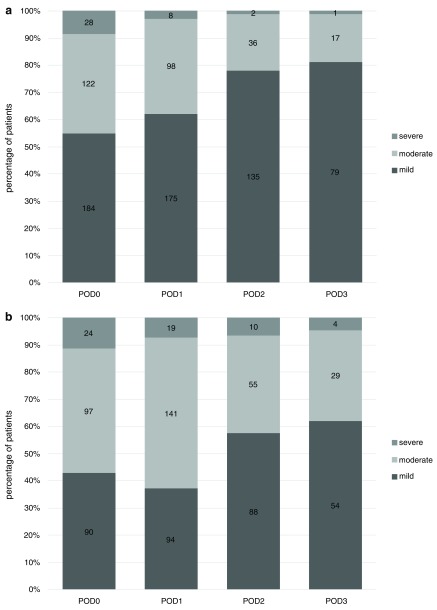
Pain after VATS for day of operation through postoperative day (POD) 3, categorized in mild, moderate and severe pain. Pain at rest ( **a**) and in movement ( **b**). Percentage of patients with NRS scores available. Absolute numbers are shown in the centre of bars. Patient discharge and missing data cause diminishing absolute numbers during progression of postoperative course.

At rest, pain was rated as mild, with median NRS rated 3-3-1-1 for POD 0 to 3. Assessed during movement, pain was rated moderate during POD 0 and 1 and mild the remaining days (median NRS 4-4-3-3 for POD 0 to 3). The proportion of patients exhibiting mild pain at rest increased from 55% on POD 0 to 81% on POD 3. The percentage of patients experiencing severe pain at rest decreased from 15% to 6% ( Figure 2).

### Pain after VATS wedge resection compared to VATS lobectomy

**Figure 3.**
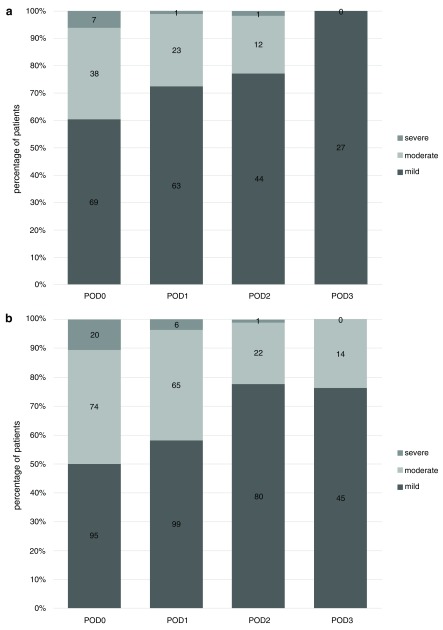
Pain at rest after VATS wedge resection ( **a**) and lobectomy ( **b**) for day of operation through postoperative day (POD) 3, categorized in mild, moderate and severe pain. Percentage of patients with NRS scores available. Absolute numbers are shown in the centre of bars.

Pain at rest after VATS wedge resection was reported as median NRS 2-2-1-1 compared to pain after lobectomy with median NRS 3-3-2-2 for POD 0 to 3. At rest, more patients were experiencing mild pain and fewer patients were experiencing severe pain after wedge resection than lobectomy all postoperative days except POD 2 where an equal percentage of patients were experiencing mild, moderate and severe pain ( Figure 3a, b). The pain pattern, mild pain versus non-mild pain, showed a difference in pain at rest for wedge resection vs. lobectomy at POD 1 (p = 0.03) and POD 3 (p = 0.006), respectively.

For POD 0 to 3 median NRS reported in movement was 3-4-3-2 after wedge resection and NRS 5-5-3-3 after lobectomy (p = 0.05 for POD 0).

### Opioid consumption

Median oxycodone consumption was 10 mg per day for POD 1 to 3 (range: 0 to 210 mg, one patient with preoperative high dose opioid treatment). Median cumulative oxycodone consumption for POD 1 to 3 was 35 mg (range: 0 to 600 mg).

**Figure 4.**
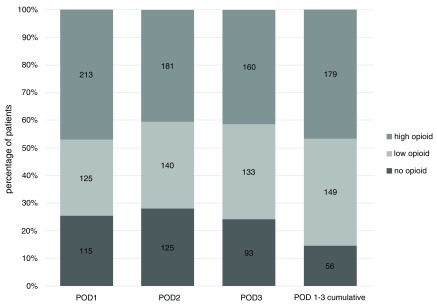
Number and percentage of patients requiring no opioid, low-dose opioid and high-dose opioid for postoperative day (POD) 1 to 3 and for total cumulative duration of POD 1 to 3. Absolute number shown inside bars. Categorized data, for definition of low and high opioid dose, see the “Statistics” section of the Methods. Cumulative opioid dose is calculated only where data is complete for all days POD 1 to 3.

After VATS, opioid treatment was not required by 25% of patients at POD 1, 28% at POD 2 and 20% at POD 3. During the first three postoperative days in total, 15% of patients did not require oxycodone at all ( Figure 4).

### Comparing opioid consumption after wedge resection and lobectomy

**Figure 5.**
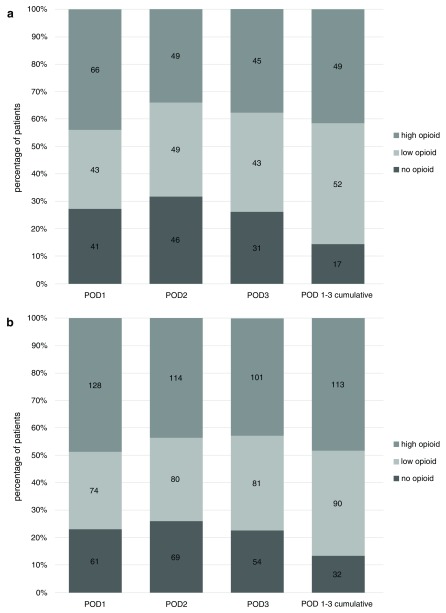
Number and percentage of patients requiring no opioid, low dose opioid and high dose opioid for postoperative day (POD) 1 to 3 and for total cumulative duration of POD 1 to 3 after video-assisted thoracoscopic surgery wedge resection ( **a**) respectively lobectomy ( **b**). Absolute number inside bars. Categorized data, for definition of low and high opioid dose see text under “statistics”. Cumulative opioid dose is calculated only where data is complete for all days POD 1 to 3.

The percentage of patients not requiring opioid POD 1 to 3 was minimally higher after VATS wedge resection. Conversely, the percentage of patients with a high opioid dose was larger for all postoperative days after lobectomy ( Figure 5a, b). Patients not needing any opioid during any of POD 1–3 was 14% for both wedge resection and lobectomy. We did not find a significant difference in oxycodone dose after wedge resection and lobectomy for POD 1 to 3 or cumulative for POD 1 to 3.

### Self-reported pain when taking age and gender into account

Older patients (>65 years of age) reported significantly less pain (mild pain vs more than mild pain) at rest (p = 0.01) and in movement (p = 0.002) at the day of surgery. Patients older than 65 had a significantly lower cumulative opioid dose during POD 1 to 3 than younger patients (median 30 vs 45 mg, p < 0.001).

Pain at rest after VATS overall was similar for women and men. POD 1, women experienced more often more than mild pain in movement after VATS (p = 0.04) and at rest after wedge resection (p = 0.04). We found no difference between sexes in cumulative oxycodone dose for any procedure.

### Adverse events

Adverse effects were rarely documented in the patient records. For 16% of the patients, symptoms of PONV were reported. No major complications related to the procedure (extrapleural catheter) were reported during the 2-year period this audit covers.

Raw patient data assessed in the present study Data include pain scores at postoperative days (POD) 0-3 at rest and with movement, alongside basic demographic information. Click here for additional data file. Copyright: © 2018 Larsson M et al. 2018 Data associated with the article are available under the terms of the Creative Commons Zero "No rights reserved" data waiver (CC0 1.0 Public domain dedication).

## Discussion

In this retrospective study we found that a surgeon placing an extrapleural catheter with continuous infusion of levobupivacaine 2.7 mg/ml at a rate of 5 ml/h was a valuable part of our multimodal pain treatment. Pain was well controlled, pain was at rest overall reported as mild and rated moderate initially and mild from POD 2 during movement. We found VATS lobectomy to be more painful than VATS wedge resection.

To achieve this level of analgesia, a median dose of only 10 mg oxycodone per day was needed. However, only 15% of our patients did not need any oxycodone at all and 47% needed more than a low dose of oxycodone during the postoperative course. This indicates that there is a potential to further improve the non-opioid part of our multimodal analgesic strategy. We did, however, see a relatively low incidence of opioid-related adverse effects, PONV was experienced in only 16% of patients.

It should be acknowledged that we used a fixed continuous infusion of 13.5 mg levobupivacaine per hour after an initial bolus of 75 mg regardless of age, body weight and surgery. To our knowledge, the optimal dose for a continuous extrapleural (or paravertebral) block is not known. A higher dose of local anaesthetic, either through higher concentration or higher rate of infusion, might offer superior analgesia. Simultaneously, the risk for adverse events due to toxicity might increase. This dose was chosen taking recommended maximal daily doses into account. Single-shot paravertebral with ropivacaine and adrenaline has been shown to decrease the systemic uptake of ropivacaine ^15^. Addition of adrenaline might allow for more local anaesthetics to be infused. Kosiński *et al.* ^11^ observed that better analgesia was achieved using a mixture of bupivacaine and adrenaline when infused through a continuous paravertebral block rather than continuous epidural, even implying an additive effect of adrenaline itself. Future trials should address systemic absorption and toxicity for continuous blocks, measuring plasma concentrations of the local anaesthetic used.

Another possibility to increase the analgesic effect of our extrapleural catheter could be to add adjuncts other than adrenaline to the local anaesthetic infused. In a prospective study, Xu *et al.* ^16^ showed that adding dexmedetomidine to a single-shot paravertebral block with ropivacaine resulted in better analgesia from 8 to 48 hours after VATS. For patients with unilateral multiple rib fractures, Mohta *et al.* ^17^ showed equal pain relief with a lower dose of ropivacaine when a continuous paravertebral block contained a low dose of fentanyl in addition to ropivacaine and adrenaline. At the same time, Bauer *et al.* ^18^ did not show an additional analgesic effect of sufentanil added to ropivacaine in a continuous paravertebral block. Whether a surgical extrapleural continuous block in VATS combining local anaesthetics, adrenaline and a short-acting opioid is superior to a block with only local anaesthetics is, to our knowledge, unknown.

We used levobupivacaine for our block. It is possible that alternative local anaesthetics offer better analgesia. Lidocaine may be an alternative having both a local block effect as well as systemic anti-inflammatory and possibly analgesic properties ^19– 21^.

This study shows also that different procedures with a difference in trauma applied cause a varying intensity in pain. However, we did not find a significant difference in opioid requirement for different procedures, even though fewer patients needed a high oxycodone dose after VATS wedge resection. Still, tailored analgesia with different types of regional analgesia or different contents infused may be the way to offer patients the optimal balance between analgesia and side-effects, which may improve postoperative recovery.

There are weaknesses in our study. This is a retrospective patient-record-based study, and all patients operated during the period were included. The wide range of oxycodone dose (0-210 mg/day) may be a result of our retrospective approach. Patients with a preoperative history of chronic pain and/or ongoing opioid treatment were not specifically identified. We used 5-mg increments in oral oxycodone dose. Using a patient-controlled analgesia regimen might have shown a more accurate opioid requirement. It should also be acknowledged that several patients were discharged before POD 3. It is not unlikely that patients discharged earlier and lost to follow-up could have experienced the least pain and least need for opioid treatment. This might skew our results towards higher median pain and higher opioid requirement.

## Conclusion

We found that a continuous surgeon-placed extrapleural catheter block to be a valuable and seemingly safe addition to our multimodal procedure specific analgesia after VATS. Whether the efficacy of the block can be improved by increasing local anaesthetic and/or adding adjuncts warrants further investigation.

## Data availability

The data referenced by this article are under copyright with the following copyright statement: Copyright: © 2018 Larsson M et al.

Data associated with the article are available under the terms of the Creative Commons Zero "No rights reserved" data waiver (CC0 1.0 Public domain dedication). 

**Dataset 1. Raw patient data assessed in the present study.** Data include pain scores at postoperative days (POD) 0–3 at rest and with movement, alongside basic demographic information. DOI: https://doi.org/10.5256/f1000research.16857.d224364 ^22^.

## References

[1] Bendixen M Jørgensen OD Kronborg C: Postoperative pain and quality of life after lobectomy via video-assisted thoracoscopic surgery or anterolateral thoracotomy for early stage lung cancer: a randomised controlled trial. *Lancet Oncol.* 2016;17(6):836–44. 10.1016/S1470-2045(16)00173-X 27160473

[2] Landreneau RJ Hazelrigg SR Mack MJ: Postoperative pain-related morbidity: video-assisted thoracic surgery versus thoracotomy. *Ann Thorac Surg.* 1993;56(6):1285–9. 10.1016/0003-4975(93)90667-7 8267426

[3] Wildgaard K Ringsted TK Hansen HJ: Persistent postsurgical pain after video-assisted thoracic surgery--an observational study. *Acta Anaesthesiol Scand.* 2016;60(5):650–8. 10.1111/aas.12681 26792257

[4] Tsubokawa N Harada H Takenaka C: Comparison of Postoperative Pain after Different Thoracic Surgery Approaches as Measured by Electrical Stimulation. *Thorac Cardiovasc Surg.* 2015;63(6):519–25. 10.1055/s-0035-1546427 25768027

[5] Davies RG Myles PS Graham JM: A comparison of the analgesic efficacy and side-effects of paravertebral *vs* epidural blockade for thoracotomy--a systematic review and meta-analysis of randomized trials. *Br J Anaesth.* 2006;96(4):418–26. 10.1093/bja/ael020 16476698

[6] Scarci M Joshi A Attia R: In patients undergoing thoracic surgery is paravertebral block as effective as epidural analgesia for pain management? *Interact Cardiovasc Thorac Surg.* 2010;10(1):92–6. 10.1510/icvts.2009.221127 19854794

[7] Baidya DK Khanna P Maitra S: Analgesic efficacy and safety of thoracic paravertebral and epidural analgesia for thoracic surgery: a systematic review and meta-analysis. *Interact Cardiovasc Thorac Surg.* 2014;18(5):626–35. 10.1093/icvts/ivt551 24488821

[8] Yeung JH Gates S Naidu BV: Paravertebral block versus thoracic epidural for patients undergoing thoracotomy. *Cochrane Database Syst Rev.* 2016;2:CD009121. 10.1002/14651858.CD009121.pub2 26897642 PMC7151756

[9] Norum HM Breivik H: Learning from the past for the present: paravertebral blocks for thoracic surgery are not without risk. *Eur J Anaesthesiol.* 2011;28(7):544–5. 10.1097/EJA.0b013e328344d953 21666545

[10] Steinthorsdottir KJ Wildgaard L Hansen HJ: Regional analgesia for video-assisted thoracic surgery: a systematic review. *Eur J Cardiothorac Surg.* 2014;45(6):959–66. 10.1093/ejcts/ezt525 24288340

[11] Kosiński S Fryźlewicz E Wiłkojć M: Comparison of continuous epidural block and continuous paravertebral block in postoperative analgaesia after video-assisted thoracoscopic surgery lobectomy: a randomised, non-inferiority trial. *Anaesthesiol Intensive Ther.* 2016;48(5):280–7. 10.5603/AIT.2016.0059 28000203

[12] Hutchins J Sanchez J Andrade R: Ultrasound-Guided Paravertebral Catheter Versus Intercostal Blocks for Postoperative Pain Control in Video-Assisted Thoracoscopic Surgery: A Prospective Randomized Trial. *J Cardiothorac Vasc Anesth.* 2017;31(2):458–63. 10.1053/j.jvca.2016.08.010 27810407

[13] Breivik H Borchgrevink PC Allen SM: Assessment of pain. *Br J Anaesth.* 2008;101(1):17–24. 10.1093/bja/aen103 18487245

[14] Gerbershagen HJ Rothaug J Kalkman CJ: Determination of moderate-to-severe postoperative pain on the numeric rating scale: a cut-off point analysis applying four different methods. *Br J Anaesth.* 2011;107(4):619–26. 10.1093/bja/aer195 21724620

[15] Karmakar MK Ho AM Law BK: Arterial and venous pharmacokinetics of ropivacaine with and without epinephrine after thoracic paravertebral block. * Anesthesiology.* 2005;103(4):704–11. 10.1097/00000542-200510000-00008 16192762

[16] Xu J Yang X Hu X: Multilevel Thoracic Paravertebral Block Using Ropivacaine With/Without Dexmedetomidine in Video-Assisted Thoracoscopic Surgery. *J Cardiothorac Vasc Anesth.* 2018;32(1):318–24. 10.1053/j.jvca.2017.06.023 29191649

[17] Mohta M Ophrii EL Sethi AK: Continuous paravertebral infusion of ropivacaine with or without fentanyl for pain relief in unilateral multiple fractured ribs. *Indian J Anaesth.* 2013;57(6):555–61. 10.4103/0019-5049.123327 24403614 PMC3883389

[18] Bauer C Pavlakovic I Mercier C: Adding sufentanil to ropivacaine in continuous thoracic paravertebral block fails to improve analgesia after video-assisted thoracic surgery: A randomised controlled trial. *Eur J Anaesthesiol.* 2018;35(10):766–73. 10.1097/EJA.0000000000000777 29373333

[19] Hollmann MW Durieux ME: Local anesthetics and the inflammatory response: a new therapeutic indication? *Anesthesiology.* 2000;93(3):858–75. 10969322 10.1097/00000542-200009000-00038

[20] McCarthy GC Megalla SA Habib AS: Impact of intravenous lidocaine infusion on postoperative analgesia and recovery from surgery: a systematic review of randomized controlled trials. *Drugs.* 2010;70(9):1149–63. 10.2165/10898560-000000000-00000 20518581

[21] Weibel S Jelting Y Pace NL: Continuous intravenous perioperative lidocaine infusion for postoperative pain and recovery in adults. *Cochrane Database Syst Rev.* 2018;6:CD009642. 10.1002/14651858.CD009642.pub3 29864216 PMC6513586

[22] Jakobsson JG Larsson M Sartipy U: Dataset 1 in: Continuous surgical multi-level extrapleural block for video-assisted thoracoscopic surgery: a retrospective study assessing its efficacy as pain relief following lobectomy and wedge resection. *F1000Research.* 2018 10.5256/f1000research.16857.d224364 PMC6468736 31031964

