# Supplementary material for: Continuous surgical multi-level extrapleural block for video-assisted thoracoscopic surgery: a retrospective study assessing its efficacy as pain relief following lobectomy and wedge resection
Source: F1000Res. 2018 Nov 12;7:1783. [Version 1] doi: 10.12688/f1000research.16857.1 (PMC6468736; doi:10.12688/f1000research.16857.1)
Supplement: Peer review history

# Peer review: Reviewer response for version 1

**DOI:** 10.5256/f1000research.18429.r45938

**Published:** 2019-04-15

**Authors:** Waldemar Gozdzik

**Affiliations:** 1 Department of Anaesthesiology and Intensive Therapy, Wroclaw Medical University, Wrocław, Poland

In this retrospective patient-record-based study, Larsson and colleagues present the analysis of pain treatment quality in 454 patients who received a continuous surgical multi-level extrapleural block for video-assisted thoracoscopic surgery.

 All patients who underwent VATS and received a continuous extrapleural block were reviewed (150 wedge resections, 264 lobectomies, and 40 miscellaneous cases). The subject taken up by the authors is undoubtedly interesting. This is an important voice in terms of quality and ideal management of postoperative pain after thoracic surgery. The authors have shown the use of an analgesia method with a potentially lower risk of complications than EDA and that the paravertebral block is effective, and satisfactory results could be obtained in this group of patients regardless of the scope of VATS operation.

 The method of analgesia used by the authors is undoubtedly safe and satisfactory, which is confirmed by the results presented, but for obvious reasons, it is not perfect. The described method of anesthesia in VATS operations is, according to the authors' opinion, the standard conduct in their center since the 2014 year.

 In the authors’ study, an initial bolus of 75 mg levobupivacaine was followed by an additional bolus of mepivacaine 100 mg at the end of surgery. An elastomeric pump infusing levobupivacaine 2.7 mg/ml at a rate of 5 ml / h (13.5 mg / h) was connected to the catheter and continued for up to 3 days. This dose was chosen for the maximal daily checks.

 Reading the text raises some questions, for example, whether there have been attempts to modify this method of analgesia in their center. Also interesting was the comment on the quality of this method of anesthesia in comparison with the methods used earlier (if it was used, for example, TEDA). Because patients underwent major thoracic surgery, chest tube removal timing seems very short. It would be interesting to know which criteria the authors followed to safely remove the chest tubes, as the authors did not specify them in their otherwise very good paper.

 The authors addressed the limitations of their study well, including a wide range of oxycodone dose, and no PCA method used, lost to follow-up in patients discharged earlier before the third post-operative day. As mentioned by the authors, it is difficult to compare their study to those available in literature because of differences in technique and study design.

   

 In conclusion: the paper is well written, and the presented data are conclusive. This is an important and valuable work dealing with difficult issues of optimal postoperative analgesia in patients after major thoracic surgery.

I have read this submission. I believe that I have an appropriate level of expertise to confirm that it is of an acceptable scientific standard.

# Peer review: Reviewer response for version 1

**DOI:** 10.5256/f1000research.18429.r43646

**Published:** 2019-02-13

**Authors:** Colin F. Royse

**Affiliations:** 1 Department of Surgery, University of Melbourne, Parkville, Victoria, Australia

The authors have conducted a retrospective review of a large sample of patients undergoing VATS with surgeon placed extrapleural catheters. The study is well done and well reported. They found that most patients had mild pain only, and only 15% had severe pain, which diminished to 6% by day 3 – suggesting good analgesic efficacy of the technique.

 I have no major problems with the methods of the paper, and it is well written.

 They did find a difference between VATS lobectomy compared to other VATS, but the authors have not offered any potential reason for this. It is possible that the surgeons place more intercostal catheters in lobectomy patients, which could increase stimulation beyond the analgesic boundary of the extrapleural catheter?

 Further in the VATS group, how many patients were included who had a pleurodesis? It is possible that the inflammation produced by pleurodesis could cause stimulation outside of the extrapleural block, and therefore requires additional pain relief.

I have read this submission. I believe that I have an appropriate level of expertise to confirm that it is of an acceptable scientific standard.
